# A New Selective PPARγ Modulator Inhibits Triglycerides Accumulation during Murine Adipocytes’ and Human Adipose-Derived Mesenchymal Stem Cells Differentiation

**DOI:** 10.3390/ijms21124415

**Published:** 2020-06-21

**Authors:** Ghina Al Haj, Federica Rey, Toniella Giallongo, Mattia Colli, Barbara Marzani, Giammaria Giuliani, Alfredo Gorio, Gian Vicenzo Zuccotti, Anna Maria Di Giulio, Stephana Carelli

**Affiliations:** 1 Department of Health Sciences, University of Milan, Via Antonio di Rudinì 8, 20142 Milan, Italy; ghina.alhaj@unimi.it (G.A.H.); federica.rey@unimi.it (F.R.); toniella.giallongo@unimi.it (T.G.); mattiacolli@shbclinic.com (M.C.); alfredo.gorio@unimi.it (A.G.); 2 Research and Development, Giuliani SpA, Via Pelagio Palagi, 2, 20129 Milan, Italy; bmarzani@giulianipharma.com (B.M.); ggiuliani@giulianipharma.com (G.G.); 3 Department of Biomedical and Clinical Sciences, University of Milan, Via G.B. Grassi 74, 20157 Milan, Italy; gianvincenzo.zuccotti@unimi.it; 4 Pediatric Research Center “Romeo ed Enrica Invernizzi”, University of Milan, Via G.B. Grassi 74, 20157 Milan, Italy

**Keywords:** adipocytes, differentiation, lipogenesis, *PPAR γ* modulator, adipogenesis

## Abstract

Understanding the molecular basis of adipogenesis is vital to identify new therapeutic targets to improve anti-obesity drugs. The adipogenic process could be a new target in the management of this disease. Our aim was to evaluate the effect of GMG-43AC, a selective peroxisome proliferator-activated receptor γ *(PPARγ)* modulator, during adipose differentiation of murine pre-adipocytes and human Adipose Derived Stem Cells (hADSCs). We differentiated 3T3-L1 cells and primary hADSCs in the presence of various doses of GMG-43AC and evaluated the differentiation efficiency measuring lipid accumulation, the expression of specific differentiation markers and the quantification of accumulated triglycerides. The treatment with GMG-43AC is not toxic as shown by cell viability assessments after the treatments. Our findings demonstrate the inhibition of lipid accumulation and the significant decrease in the expression of adipocyte-specific genes, such as *PPARγ*, *FABP-4*, and *leptin*. This effect was long lasting, as the removal of GMG-43AC from culture medium did not allow the restoration of adipogenic process. The above actions were confirmed in hADSCs exposed to adipogenic stimuli. Together, these results indicate that GMG-43AC efficiently inhibits adipocytes differentiation in murine and human cells, suggesting its possible function in the reversal of adipogenesis and modulation of lipolysis.

## 1. Introduction

Adipogenesis is a complex process which includes the integration of many different signalling pathways and transcription factors [1]. Understanding the adipogenesis process could be of key importance for the development of therapeutic strategies for obesity [2]. Various medicinal plants have been tested for their inhibitory effects on adipogenesis such as phenolic acids, flavonoids, stilbenes, and lignans [3,4]. For example, a study from Lagouge et al. showed that mice fed a high fat diet with supplemented resveratrol (3,4,5-trihydroxy-trans-stilbene) increases mitochondrial content/activity in skeletal muscle brown adipose tissue and the liver to protect against developing diet-induced obesity and improving metabolic disturbances [5]. Adipogenesis begins with a common multipotent precursor cell, that progressively goes through four sequential phases: pre-confluent proliferation, growth arrest, mitotic clonal expansion, and terminal differentiation [6,7,8,9,10]. The differentiation of preadipocytes into adipocytes is accompanied by alterations in gene expression and protein synthesis [11,12]. Mitotic clonal expansion is accompanied by the induction of *CCAAT*/enhancer-binding protein *(C/EBP) β* and *δ*. These factors are rapidly expressed upon hormonal induction and then could transcriptionally activate the *C/EBPα* and peroxisome proliferator-activated receptor γ (*PPAR γ*), which are the actors during the terminal phase of differentiation [13]. All of them are necessary for the expression of adipocyte-specific genes, such as fatty acid binding protein (*FABP-4*), leptin, lipoprotein lipase, and others [11].

3T3-L1 preadipocytes are the most used in vitro cell line for investigating adipogenesis [10]. These cells are clonally isolated from Swiss 3T3 cells derived from 17–19-day mouse embryos. They display a fibroblast-like morphology that, under appropriate conditions, can acquire an adipocyte-like phenotype [6,8,9]. They differentiate spontaneously into adipocytes when exposed to a hormonal cocktail composed of dexamethasone (DEX), 3-isobutyl-1-methylxantine (IBMX), and high concentrations of insulin [6,8,9]. During adipocyte differentiation cell morphology, cytoskeletal components and the level and type of extracellular matrix components (ECM) change as well [6,9,11].

Mesenchymal stem cells (MSCs) are multipotent cells that have the capability into differentiating into various cell types, such as osteoblasts, chondrocytes, and adipocytes [14]. MSCs can be successfully derived from different tissues such as skin, bone, cartilage, and adipose tissue [15]. These cells are commonly used in regenerative medicine for the treatment of damaged tissue [16]. Human adipose derived mesenchymal stem cells (hADSCs), are derived from MSCs derived from the adipose tissue [17]. Similar to 3T3-L1 cells, they can be differentiated into adipocytes when exposed to the same hormonal cocktail used for 3T3-L1 differentiation.

GMG-43AC is an experimental drug presenting with a structure derived from propionic acid (PA). PA is fermented in the colon by microbiota. It can reach the blood and adipose tissue where it can decrease fatty acids in plasma by the inhibition of lipolysis and induce lipogenesis and suppress fatty acid production in the liver [18,19,20]. This short-chain fatty acid molecule has been shown to inhibit lipolysis and de novo lipogenesis in SZ95 human sebocytes [18,21,22]. Its mechanism of action is yet to be fully eviscerated, but some previous studies have highlighted its function in multiple cellular contexts. Specifically, Ramot and colleagues evaluated the GMG-43AC effects on normal human keratinocytes (NHK) cell lines. In this context, its function as a *PPARγ* modulator was demonstrated. Another study from Mastrofrancesco and colleagues, investigated the effects of GMG-43AC in SZ95 sebocytes cell line as experimental model for acne [23]. The molecule seems to counteract several processes, including sebaceous lipogenesis, inflammation, alteration of lipid composition, and cellular proliferation [23]. On the contrary, other studies on this compound found that PPAR*γ* modulation resulted in increased lipid accumulation in rat preputial sebocytes and in human sebocytes [21,24] as evidence on the GMG-43AC mechanism of action was scarce and its effects on sebogenesis is not completely clear. Even so, the specific role that GMG-43AC plays on adipocytes differentiation is still unknown and controversial. The aim of this work was thus to investigate the potential effects of this compound on the murine 3T3 L1 and hADSCs adipocyte differentiation process, through an investigation of the molecular pathways involved. 

## 2. Results

### 2.1. GMG-43AC Inhibits Triglycerides Accumulation in Murine 3T3-L1 Cells

**Figure 1:**
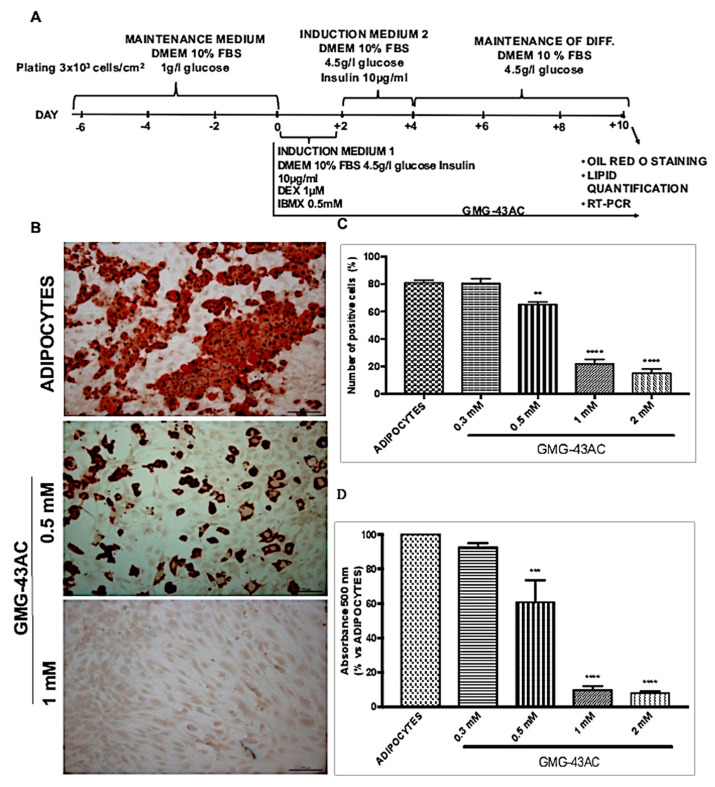
Effect of GMG-43AC on lipid accumulation in 3T3-L1 adipocytes. (**A**) Two-day post-confluent (day 0) 3T3-L1 preadipocytes were induced to differentiate in the presence of GMG-43AC of increasing concentrations for 10 days. The assays were performed on day 10. Intracellular lipids were stained with Oil Red O. (**B**) Oil Red O staining of adipocytes (top), cells treated with 0.5 mM (middle) and 1 mM GMG-43AC (bottom) at day 10. (**C**) Percentage of Oil Red O—positive cells in reference to total cell population. Reported values (mean ± SEM) are the result of 3 independent experiments and for each experiment at least 3 independent fields were considered for every condition (*n* = 9, ** *p* < 0.01, **** *p* < 0.0001 vs. adipocytes). (**D**) The level of accumulated triglycerides labelled with Oil Red O in 3T3-L1 derived adipocytes and cells treated with different GMG-43AC doses was spectrophotometrically determined at 500 nm at day 10. Each experimental condition was assayed in triplicate and the graph refers to the mean of 3 independent experiments (*n* = 9, *** *p* < 0.001, **** *p* < 0.0001 vs. adipocytes).

To investigate the effects of GMG-43AC on adipocyte differentiation, we evaluated the triglyceride accumulation by means of Oil Red-O staining. Briefly, two days post-confluent 3T3-L1 preadipocytes were treated with a cocktail of inducing agents: dexamethasone (DEX) and 3-isobutyl-1-methyl xanthine (IBMX) in the presence of fetal bovine serum (FBS) and insulin (Figure 1A,B see Material and Methods for further details). After 10 days of differentiation, 3T3-L1 adipocytes accumulate lipid droplets in huge amounts (Figure 1B). The quantification of Oil Red-O positive cells gave evidence that 80.5 ± 2.0% accumulated triglycerides (Figure 1C). The effect of GMG-43AC was assayed by the administration of the compound at day 0 of differentiation protocol (Figure 1A). Low concentrations of GMG-43AC (ranging from 0.1 µM to less than 0.3 mM) had no significant inhibitory effects on lipid accumulation after 10 days of treatment (data not shown) and were not taken into consideration for further experiments. Significant inhibitory effects were observed with GMG-43AC concentrations of 0.5 mM and higher (Figure 1B–D). The higher GMG-43AC dosage (1 mM and 2 mM) reduced by three folds the number of cells positive to Oil Red-O staining (Figure 1C). The quantification of lipid accumulation obtained by measuring the absorbance at 500 nm after the extraction of the triglycerides stained with Oil Red-O further confirmed these observations (Figure 1D). These results suggest that GMG-43AC inhibited lipid accumulation in 3T3-L1 adipocytes in a dose-dependent manner starting from the 0.5 mM dosage. The effect of GMG-43AC on cellular viability and apoptosis was also evaluated.

### 2.2. GMG-43AC Does Not Influence Viability of 3T3-L1 Cells

**Figure 2:**
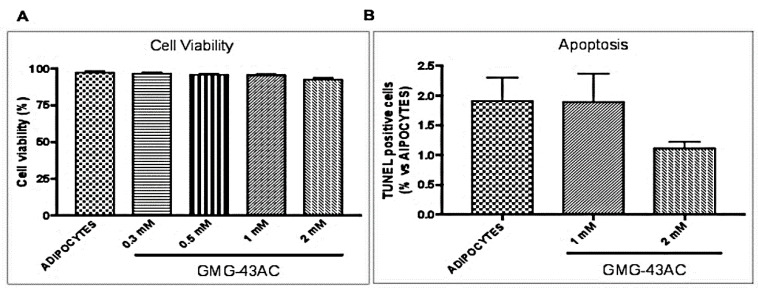
GMG-43AC does not induce apoptosis in 3T3-L1 cells. (**A**) Effect of GMG-43AC on the viability of 3T3-L1 cells was determined by Propidium Iodide Staining. Values are expressed as a percentage of propidium iodide positive cells (cell viability) after a 10 day incubation (*n* = 5). (**B**) Control (adipocytes) and GMG-43AC-treated cells were fixed and analyzed for DNA fragmentation by means of TUNEL(Terminal deoxynucleotidyl transferase dUTP Nick End Labeling) after 10 days of differentiation. Percentage of TUNEL-positive cells in reference to total cell population. Data are expressed as the mean ± SEM of two independent experiments, and for each experiment four fields were considered for each condition (*n* = 8).

The effect of GMG-43AC on cellular viability and apoptosis was also evaluated. After the treatment, cell viability was determined by propidium iodide incorporation and it was demonstrated that GMG-43AC did not influence cell viability, even at the highest dosages (2 mM) (Figure 2A). Apoptosis was studied by means of TUNEL (terminal deoxynucleotidyl transferase dUTP nick end labeling) assay and the results show that the number of TUNEL positive cells was comparable to that of adipocytes sample. The number of apoptotic nuclei was very low and reached a maximum of 2 percent (Figure 2B), demonstrating the lack of toxicity induced by the drug at dosages up to 2 mM.

### 2.3. GMG-43AC Influences the Expression of Early Adipocytes Markers

**Figure 3:**
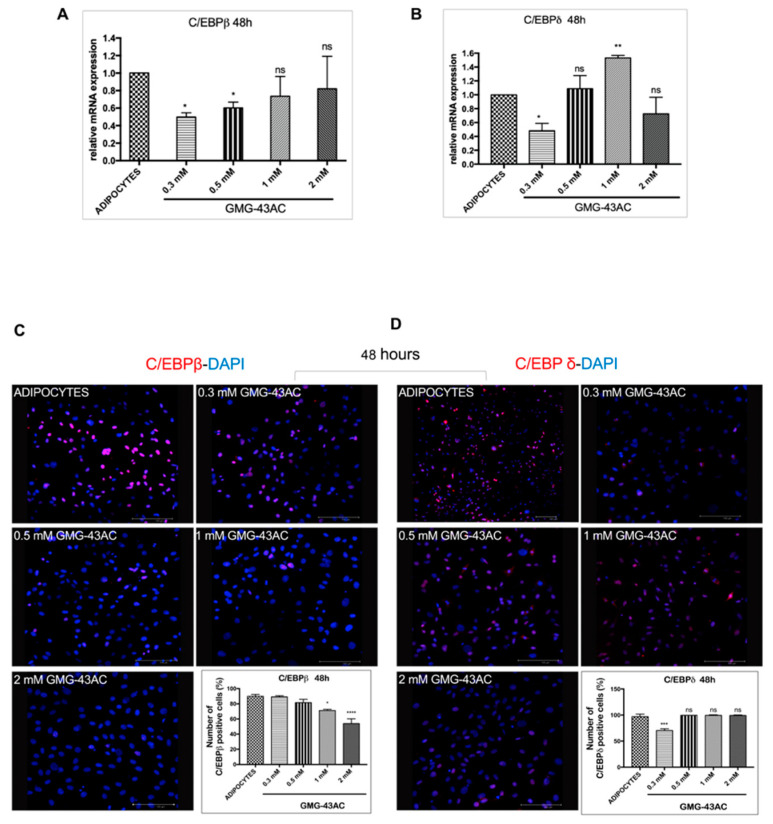
Effect of GMG-43AC on the expression of early adipogenesis transcription factors. Two-day post-confluent 3T3-L1 cells were induced to differentiate in the presence of different GMG-43AC doses and were lysed at the indicated times for subsequent analysis. (**A**) The mRNA expression levels of *C/EBPβ*. (**B**) *C/EBPδ* were evaluated 48 h after the induction of adipogenesis by means of Real Time PCR. Results were normalized to *18S rRNA* and data are expressed as mean ± SEM (*n* = 4). (**C**) Immunofluorescence analysis of *C/EBPβ* and (**D**) *C/EBPδ* distribution and localization. Nuclei were stained with *DAPI* (blue). Reported values (mean ± SEM) are the result of three independent experiments, and for every experiment three fields were considered for each condition (*n* = 9, * *p* < 0.05, ** *p* < 0.01, *** *p* < 0.001, **** *p* < 0.0001 vs. adipocytes).

To examine the effect of GMG-43AC on the expression of factors involved in early adipogenesis regulation, preadipocytes were induced to differentiate for 48 h in the presence or absence of GMG-43AC and harvested at the end of the observational period. The isoforms *C/EBPβ* and *δ* are two critical markers for the early phase of differentiation and are dramatically up-regulated within the first hours after induction of adipogenesis by IBMX and DEXA [6]. They are known to have a crucial role in the regulation of *PPARγ* and *C/EBPα* expression [25]. The data shows that after 48 h of adipogenic differentiation, drug dosages seem to influence mRNA levels of both C/*EBPβ* (Figure 3A) and *C/EBPδ* (Figure 3C). The expression of *C/EBPβ* at 48 h shows no significance by higher dosages of the drug (1 mM and 2 mM). Lower dosages of the drug (0.3 mM and 0.5 mM) also significantly decrease *C/EBPβ’s* expression (Figure 3A). The immunofluorescence analysis also shows that at 48 h *C/EBPβ* and *C/EBPδ* localized in the nucleus as expected (Figure 3C,D). The discrepancies in *C/EBPβ’s* protein’s expression with respect to the mRNA levels could be due to an impairment on protein’s expression rather than the mRNA transcription process. Indeed, a compensatory increase in translation of these mRNA could be present, providing a possible explanation for the lack of differences in protein expression. 

### 2.4. GMG-43AC Down-Regulates the Expression of Late Markers of Adipose Differentiation

**Figure 4:**
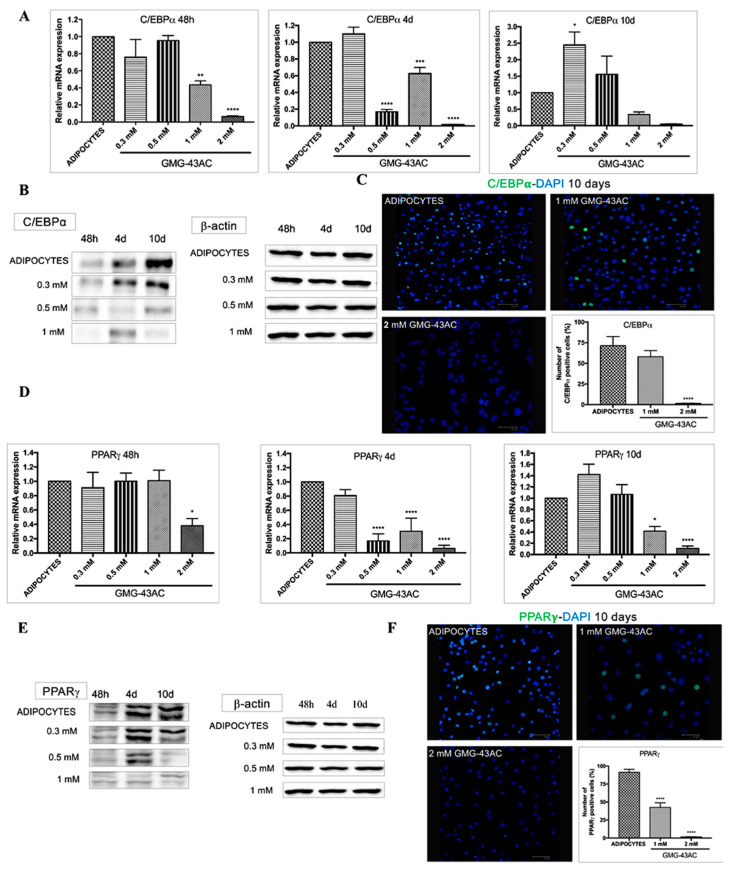
Effect of GMG-43AC on the expression of adipogenesis transcription factors. Adipocytes were induced to differentiate with different concentrations of GMG-43AC and harvested at indicated time during the differentiation period. (**A**) The mRNA expression of *C/EBPα* was analyzed by Real Time PCR. Results were normalized to *18S rRNA* and data are expressed as mean ± SEM (*n* = 4, * *p* < 0.05, ** *p* < 0.01, *** *p* < 0.001, **** *p* < 0.0001 vs. adipocytes). (**B**) Western blotting analysis of *β-Actin* and *C/EBP*α involved in adipogenesis (**C**) Immunofluorescence analysis of *C/EBP*α distribution and localization. Nuclei were stained with *DAPI* (blue). Reported values (mean ± SEM) are the result of three independent experiments, and for every experiment two fields were considered for each condition (*n* = 6, *** *p* < 0.001 vs. adipocytes). (**D**) The mRNA expression of *PPARγ* was analyzed by Real Time PCR. Results were normalized to *18S rRNA* and data are expressed as mean ± SEM (*n* = 4, * *p* < 0.05, **** *p* < 0.0001 vs. adipocytes). (**E**) Western blotting analysis of *β-Actin* and *PPAR*γ in adipogenesis. (**F**) Immunofluorescence analysis of *PPARγ* distribution and localization. Nuclei were stained with *DAPI* (blue). Reported values (mean ± SEM) are the result of three independent experiments and for every experiment 2 fields were considered for each condition (*n* = 6, **** *p* < 0.0001 vs. adipocytes).

The transcription factors *C/EBPβ* and *δ*, after their rapid induction, transcriptionally activate the *C/EBPα* and *PPARγ*, which are characteristics of the terminal phase of differentiation [11]. As expected during the adipogenesis process the levels of *C/EBPα* started to increase at 48 h and were maximal at day 10 of differentiation (Figure 4A–C). GMG-43AC decreases the expression levels of this protein (Figure 4B,C). More specifically, the mRNA levels of *C/EBPα* are significantly decreased by higher doses of GMG-43AC (1 mM and 2 mM) at 48 h and after 4 days since the induction of adipogenesis. The lower doses show controversial results, with 0.5 mM leading to a decrease in *C/EBPα*’s expression levels only at the day 4 time-point, and 0.3 mM leading to a curious increase in gene expression at day 10 (Figure 4A). Western blotting was performed to analyse the protein expression of *C/EBP α* and *PPAR γ* (Figure 4B,E). The effect on the *C/EBPα* protein is similar, with a significant decrease in its level at the end of the 10 days observational period, when the cells are treated with 2 mM GMG-43AC dose at the end of the 10 days’ observational period (Figure 4D–F). *PPAR γ* mRNA levels resulted decreased, with only the 2 mM dose being effective as early as 48 h since the differentiation induction. Identically to *C/EBPα*, 1 mM GMG-43AC leads to an inhibition of *PPAR γ* at day 4 and day 10, whereas the 0.5 mM dose is effective only at the day 4 time-point (Figure 4D–F). Conversely, the 1 mM and 2 mM GMG-43AC doses also lead to the decrease of *PPAR γ* protein expression (Figure 4E,F).

### 2.5. GMG-43AC Inhibits the Expression and Modifies the Localization of Adipocyte-Specific Markers

**Figure 5:**
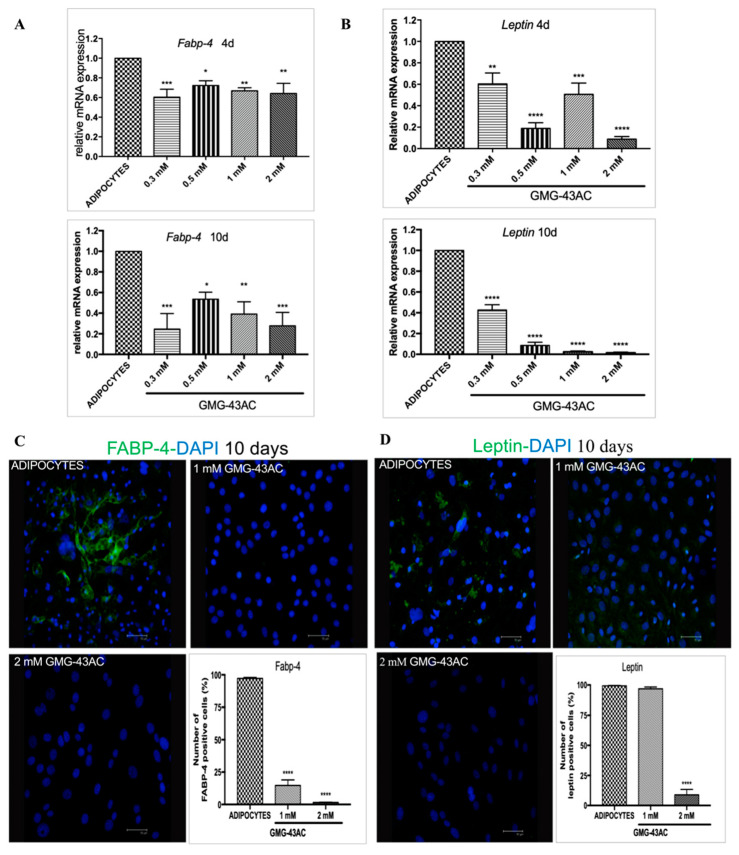
Effect of GMG-43AC on the expression and localization of adipocyte-specific markers. The mRNA expression of (**A**) *FABP-4* and (**B**) *leptin* was analyzed by Real Time PCR. Results were normalized to *18S rRNA* and data are expressed as the mean ± SEM. (*n* = 5, * *p* < 0.05, ** *p* < 0.01, *** *p* < 0.001, **** *p* < 0.0001 vs. adipocytes). Cells were fixed at day 10 of the differentiation process and stained with a specific antibody to investigate by immunofluorescence the distribution and localization of (**C**) *FABP*-4 and (**D**) *leptin*. Nuclei were stained with *DAPI* (blue). Reported values (mean ± SEM) are the result of three independent experiments, and for every experiment two fields were considered for each condition. (*n* = 6, **** *p* < 0.0001 vs. adipocytes).

*C/EBPα* and *PPAR γ* are necessary for the expression of adipocyte-specific genes, such as fatty acid binding protein (*FABP-4*), leptin, and lipoprotein lipase [9,11]. The following step was to investigate whether GMG-43AC also has an effect on these downstream molecules. As a consequence of the above outcomes, all the analysed drug doses lead to a decrease in the mRNA levels of *FABP-4* and *leptin*, present already at day 4 and maintained at day 10 (Figure 5A,B). This is maintained for the *FABP-4* protein expression (Figure 5C) whereas for Leptin the 1 mM dose did not decrease the percentage of positive cells, even if the fluorescence intensity was lower in all cells. Only the 2 mM dose seems to be effective in decreasing the protein levels (Figure 5D). However, it was noticeable that even though the dose of 1 mM did not reduce the number of positive cells, it changed the intracellular distribution of the protein, from the cytoplasm to the nucleus (Figure 5D). Our results indicated that GMG-43AC inhibited 3T3-L1 preadipocyte differentiation mostly through down-regulation of *C/EBP*α and, even more so, *PPAR γ*, leading to a subsequent downregulation of their target genes *FABP-4* and *leptin* (Figure 5). A compensatory increase in translation of these mRNA could be present, providing a possible explanation for the lack of differences in protein expression.

### 2.6. GMG-43AC Induces Lipolysis and Influences the Hsl Gene

**Figure 6:**
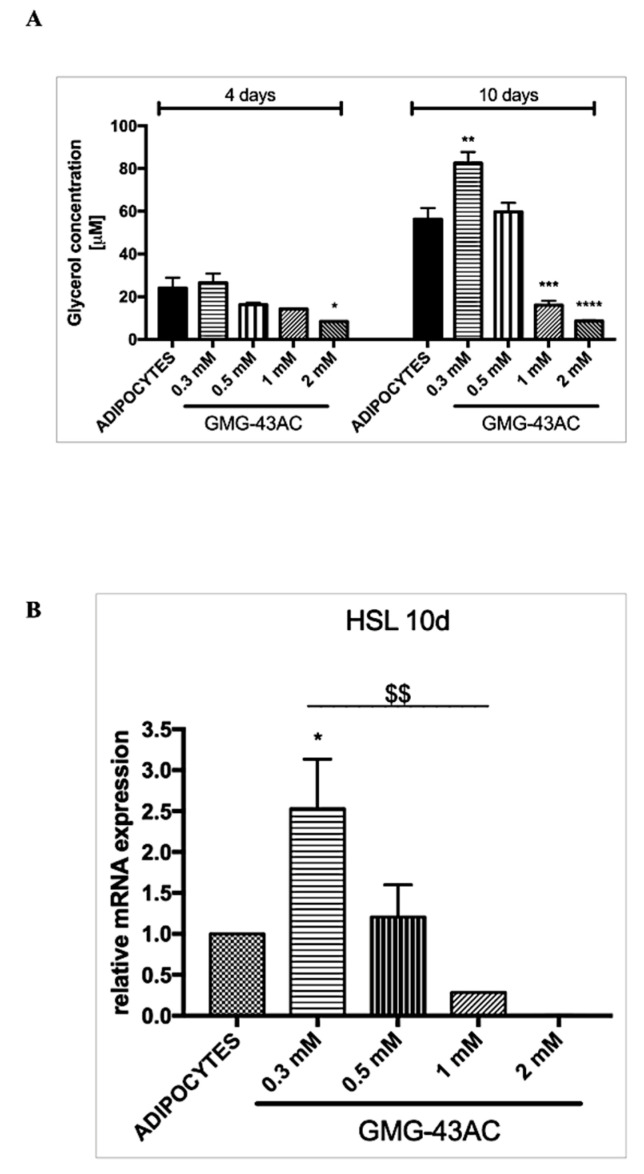
GMG-43AC stimulates lipolysis in 3T3-L1 adipocytes. (**A**) 3T3-L1 preadipocytes were treated with GMG-43AC for the indicated times and lipolysis was assessed by the amount of glycerol released into the media. Data are expressed as mean ± SEM (*n* = 3, * *p* < 0.05, ** *p* < 0.01, *** *p* < 0.001, **** *p* < 0.0001 vs. adipocytes). (**B**) Analysis of *Hsl* mRNA levels by real-time RT-PCR after 10 days of treatment with different dosages of GMG-43AC (*n* = 3, * *p* < 0.05, vs. ADIPOCYTES: $$ *p* < 0.01, vs. 0.3 mM GMG-43AC).

The activity of hormone-sensitive lipase (*Hsl*) increases during adipogenesis, with its function being the hydrolysis of stored triglycerides into free fatty acids [26]. Our data shows that the amount of glycerol released into media was higher during the adipocyte differentiation process (Figure 6A). The results obtained at 4 days of differentiation indicate that the amount of glycerol released into the media was down-regulated in a dose-dependent manner by the investigated drug, and this was even more evident at 10 days of differentiation (Figure 6A). This is associated with a reduction in *Hsl* mRNA levels caused by GMG-43AC at dosages of 1 and 2 mM (Figure 6B). There is also an increase in glycerol release and HSL expression when cells are treated with the 0.3 mM dosage.

### 2.7. Reversion of Adipogenesis Process by GMG-43AC

**Figure 7:**
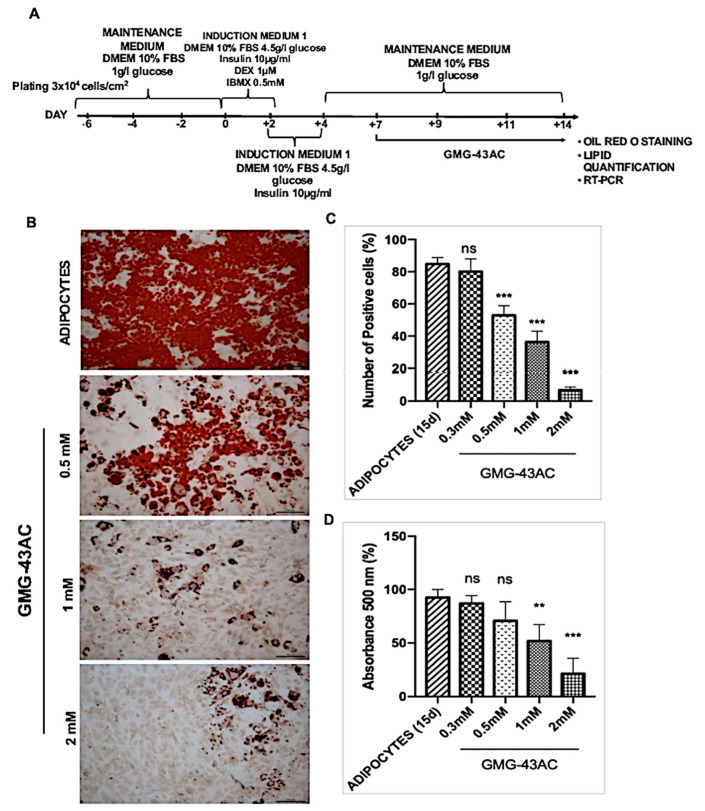
Reversion of adipogenesis process by GMG-43AC. (**A**) Cells were differentiated for 7 days and GMG-43AC was subsequently added to the culture medium and maintained for the following 7 days. (**B**) Oil Red O staining of control adipocytes and treated with three different concentrations of GMG-43AC (0.5 mM, 1 mM, 2 mM); (**C**) Percentage of positive cells in reference to total cells population. Reported values (mean ± SEM) are the result of two independent experiments and for each experiment 4 fields were considered for each condition (*n* = 8, *** *p* < 0.001 vs. ADIPOCYTES (15d)). (**D**) Levels of accumulated triglycerides (labeled with Oil Red O) in 3T3-L1 undifferentiated cells and adipocytes after 7 days reversion with GMG-43AC as evidence by quantitative absorbance at 500 nm wavelength. Each experimental condition was assayed in triplicate and the graph is referred to the means of two independent experiments. Values are reported as mean ± SEM (*n* = 8, ** *p* < 0.01, *** *p* < 0.001 vs. ADIPOCYTES 15d).

GMG-43AC was capable of promoting the loss of accumulated triglycerides by 3T3-L1 cells when fully differentiated for 7 days and then treated with the drug for 7 more days (Figure 7A). Oil Red O images showed a dose-dependent effect and significant with a dose starting from 0.5 mM (Figure 7B). The quantification of the effects of GMG-43AC on the decrease of fat cell number and total fat was shown in Figure 7C,D. The reverting effect of GMG-43AC treatment can be observed from the concentration of 0.5 mM and higher.

### 2.8. The Inhibition of Adipogenesis by GMG-43AC Is Not Reversible and Can Persist in a Long-Term Observational Period

**Figure 8:**
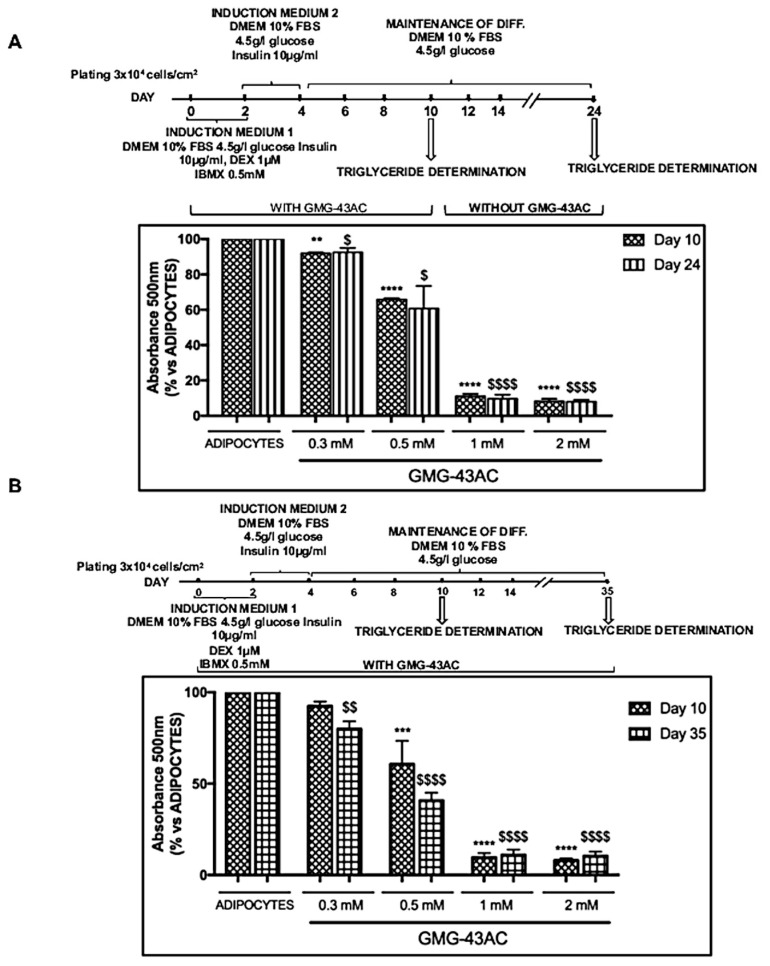
GMG-43AC inhibits adipocyte differentiation in 3T3-L1 cells in the long term. (**A**) 3T3-L1 preadipocytes were differentiated in presence of GMG-43AC (0.3–2 mM) for 10 days and then cells were incubated in the maintenance medium without GMG-43AC for the following 14 days for a total of 24 days. Levels of accumulated triglycerides were quantified by Oil Red O staining measuring the absorbance at 500 nm wavelength at Day 10 and 24. Data are expressed as mean ± SEM (*n* = 3, ** *p* < 0.01, *** *p* < 0.001, **** *p* < 0.0001 vs. adipocytes at day 10; $$ *p* < 0.01, $$$ *p* < 0.001, $$$$ *p* < 0.0001 vs. adipocytes at Day 24). (**B**) The 3T3-L1 cells were differentiated in the presence of GMG-43AC for 10 days and prolonged until 35 days. Levels of accumulated triglycerides were quantified by Oil Red O staining measuring the absorbance at 500 nm wavelength at Day 10 and 35. Each experimental condition was assayed in triplicate and the graph refers to the mean of three independent experiments. Data are expressed as mean ± SEM (*n* = 3, *** *p* < 0.001, **** *p* < 0.0001 vs. adipocytes at day 10; $ *p* < 0.05, $$ *p* < 0.01, $$$$ *p* < 0.0001 vs. adipocytes at Day 35).

To examine whether GMG-43AC inhibitory effect on adipocyte differentiation is stable, 3T3-L1 cells were differentiated in the presence of drug for 10 days, and then were incubated in the differentiation maintenance medium (DMEM, 4.5 g/L of glucose; 10% FBS) for the following 14 days without the drugs for a total of 24 days (Figure 8A). 3T3-L1 treated with GMG-43AC and incubated in the maintenance medium for 24 days did not restart to synthesize and accumulate triglycerides (Figure 8A), the accumulation of triglycerides in two experiments (10 days and 24 days) was similar. Collectively, 3T3-L1 cells differentiated in adipocytes in the presence of GMG-43AC and subsequently incubated in the maintenance medium for 14 days, did not resume to synthesize and accumulate triglycerides. Furthermore, the results suggested that GMG-43AC inhibition of triglycerides synthesis was long-lasting. Moreover, the test was prolonged up to 35 days. Our results show that not only the inhibitory effect is maintained, but also that the 0.3 mM and 0.5 mM doses become more effective in the inhibition of differentiation at 35 days than at 10 days, even if they do not reach the same level of efficacy observed when cells are treated with the highest dosages (1 mM and 2 mM) (Figure 8B).

### 2.9. GMG-43AC Inhibits Lipids Accumulation and Leads to a Loss of Accumulated Triglycerides in the Differentiation Induced by Troglitazone

**Figure 9:**
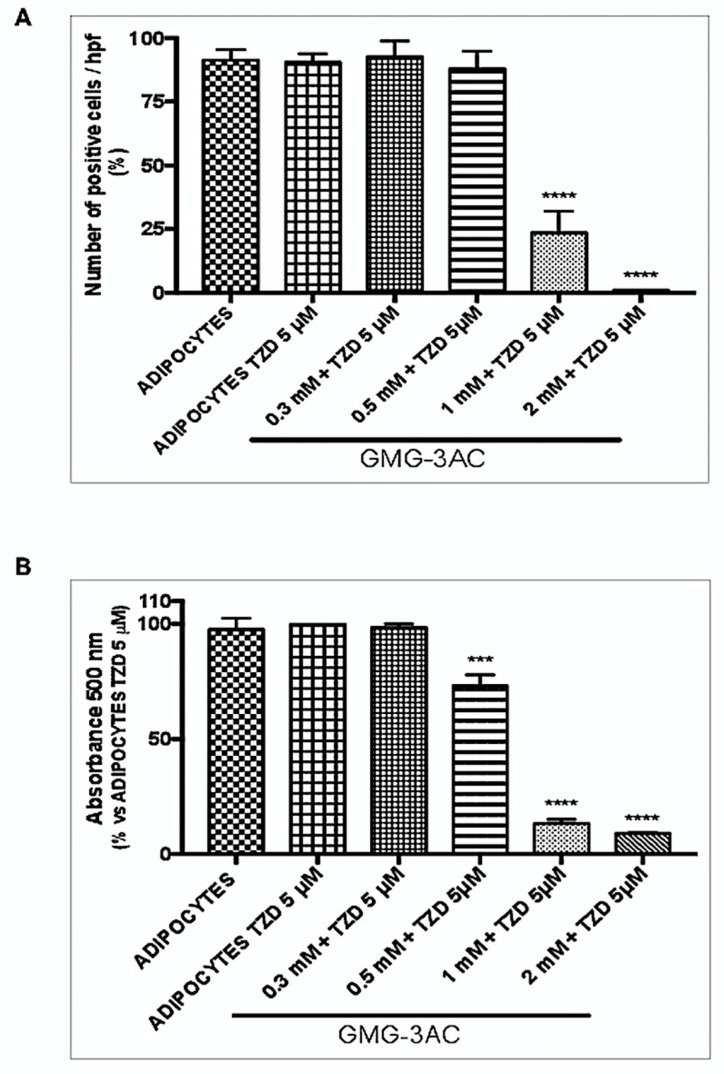
GMG-43AC inhibits lipids accumulation and promotes the loss of accumulated triglycerides in the differentiation induced by troglitazone. Two-day post confluent (day 0) 3T3-L1 preadipocytes were induced to differentiate with troglitazone (TZD) (10 μM) and insulin (10 μg/mL) in the presence of GMG-43AC of increasing concentrations for 10 days. Intracellular lipids were stained with Oil Red O. (**A**) Percentage of Oil Red O—positive cells in reference to total cell population. Reported values (mean ± SEM) are the result of three independent experiments and for each experiment at least 3 fields were considered for each condition (*n* = 9, **** *p* < 0.001 vs. adipocytes). (**B**) Quantification of accumulated triglycerides labelled with Oil Red O determined at 500 nm after Oil Red O staining. Each experimental condition was assayed in triplicate and the graphs are referred to the means of three independent experiments (*n* = 3, *** *p* < 0.001, **** *p* < 0.001 vs. adipocytes).

Troglitazone is a potent inducer of adipocyte differentiation and works by activating peroxisome proliferator-activated receptors (*PPAR*s). It is a ligand to both *PPAR γ,* and more strongly *PPARα* [27,28]. Troglitazone (TZD; 5 μM), was added to culture medium in presence of insulin (10 µg/mL), and the adipocyte differentiation was studied at day 10 [29]. GMG-43AC at a different concentration was added to the culture medium (in presence of TZD and insulin) at day 0 and maintained until the end of the differentiation. Our results show that GMC-43AC was able to counteract the synthesis and accumulation of triglycerides induced by TZD (Figure 9A,B) This inhibitory action was evident starting from the dosage 0.5 mM (Figure 9).

### 2.10. GMG-43AC Inhibits Triglycerides Accumulation and Has an Effect on the Expression of Adipocyte-Specific Genes in Human Adipose Derived Stem Cells (hADSCs)

**Figure 10:**
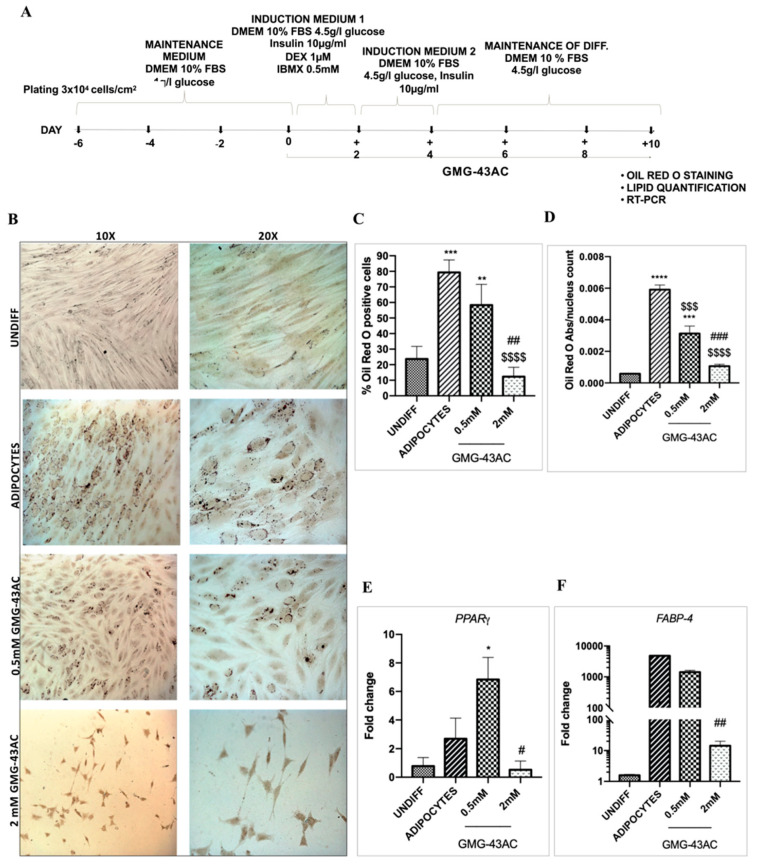
Oil Red O staining and expression of *PPARγ* and *FABP-4* during human Adipose Derived Stem Cells (hADSCs) differentiation. (**A**) hADSCs differentiation in the presence of 0.5 mM and 2 mM GMG-43AC for 10 days; (**B**) Oil Red O staining of differentiated hADSCs and treated with GMG-43AC at Day 10. hADSCs were differentiated and treated with 0.5- and 2-mM GMG-43AC for 10 days; (**C**) Percentage of positive cells in reference to the total population; (**D**) Levels of accumulated triglycerides (labelled with Oil Red O) in hADSCs undifferentiated and differentiated treated with GMG-43AC as evidenced by quantitative absorbance 500 nm wavelength (*n* = 3). (**E**) *PPARγ*; (**F**) *FABP-4*; Reported values (mean ± SEM) are the result of 3 independent experiments, and for each experiment at least three independent fields were considered for every condition (* *p* < 0.05, ** *p* < 0.01, *** *p* < 0.001, **** *p* < 0.0001 vs. UNDIFF; $$$ *p* < 0.001, $$$$ *p* < 0.0001 vs. ADIPOCYTES; # *p* < 0.05, ## *p* < 0.01, ### *p* < 0.001, vs. experimental groups).

To investigate if GMG-43AC’s effects were maintained on hADSCs, we also evaluated the accumulation of triglycerides by means of Oil Red-O staining for 10 and 14 days. Briefly, hADSCs were differentiated in the presence of 0.5 mM or 2 mM GMG-43AC for 10 days (Figure 10A). We observed a significant inhibitory effect with both 0.5 mM and 2 mM GMG-43AC concentrations on intracellular lipid accumulation (Figure 10B,C). The quantification of lipid accumulation obtained by measuring the absorbance at 500 nm after the extraction of the triglycerides stained with Oil Red-O further consolidated this finding (Figure 10D). We evaluated *PPARγ* and *FABP-4* in hADSCs and we observed that in 10-day differentiation, both *PPARγ* and *FABP-4* were highly expressed in the 0.5 mM GMG-43AC treated cells whereby extremely downregulated in the 2 mM GMG-43AC (Figure 10E,F). These results indicate that in the 10-day differentiation, the concentration of 2 mM GMG-43AC has a preventive effect of differentiation in hADSCs.

**Figure 11:**
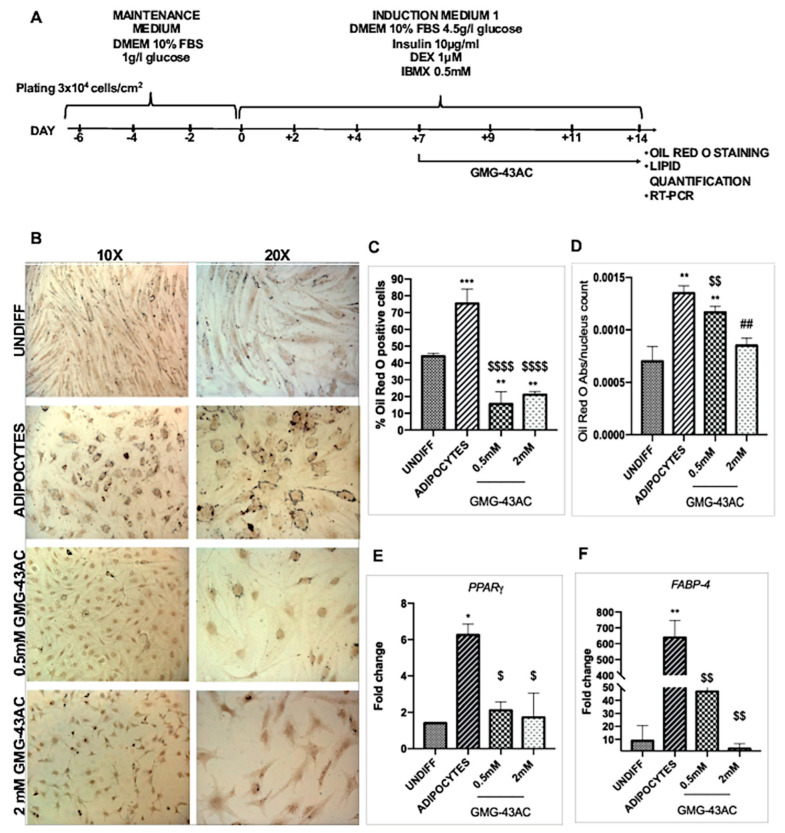
Oil Red O staining and expression of *PPARγ* and *FABP-4* in hADSCs differentiated for 14 day; (**A**) hADSCs differentiation in the presence of 0.5 mM and 2 mM GMG-43AC for 14 days; (**B**) Oil Red O staining of differentiated hADSCs and treated with GMG-43AC at Day 14. hADSCs were differentiated for 7 days and then treated with 0.5 and 2 mM GMG-43AC for 7 days; (**C**) Percentage of positive cells in reference to the total population; (**D**) Levels of accumulated triglycerides (labelled with Oil Red O) in hADSCs undifferentiated and differentiated treated with GMG-43AC as evidenced by quantitative absorbance 500 nm wavelength; (*n* = 3). (**E**) *PPARγ*; (**F**) *FABP-4*; Reported values (mean ± SEM) are the result of three independent experiments, and for each experiment at least three independent fields were considered for every condition (* *p* < 0.05, ** *p* < 0.01, *** *p* < 0.001, **** *p* < 0.0001 vs. UNDIFF $ *p* < 0.05, $$ *p* < 0.01, $$$$ *p* < 0.0001 vs. ADIPOCYTES; ## *p* < 0.01 vs. experimental groups).

**Figure 12:**
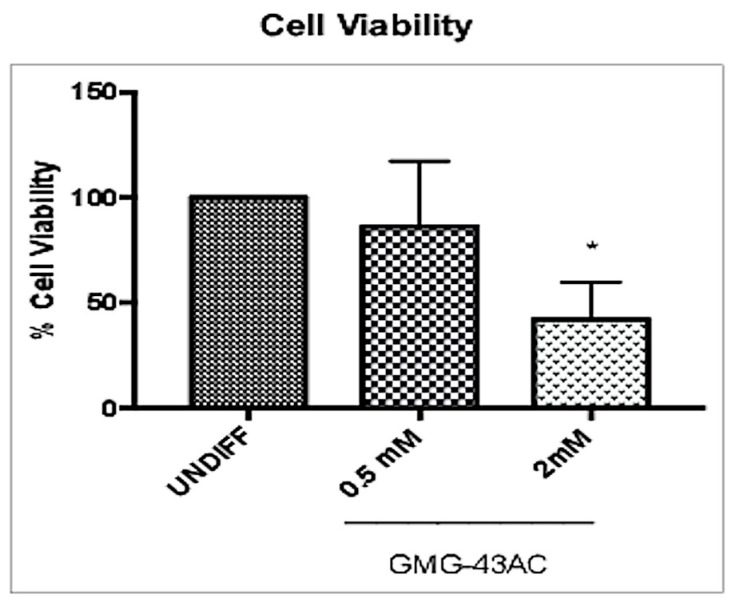
GMG-43AC halts cells proliferation in high concentration. Effect of GMG-43AC on cell proliferation of hADSCs was determined by MTT assay. Values are expressed as a percentage of the MTT absorbance of the control cells, which was set to 100%. Data are expressed as the mean ± SEM of three independent experiments (4 wells/experiment, *n* = 12; * *p* < 0.05 vs. UNDIFF).

Another group of hADSCs were differentiated for 7 days without the presence of the drug, then 7 days with the presence of 0.5 mM or 2 mM GMG-43AC for a total of 14 days (Figure 11A). Similar to the 10-day differentiation with GMG-43AC, both concentrations of GMG-43AC also had a significant inhibitory effect in the 14-day differentiation with reduced number of cells positive to Oil Red-O staining (Figure 11B,C). The quantification of lipid accumulation obtained by measuring the absorbance at 500 nm after the extraction of the triglycerides stained with Oil Red-O further consolidated this finding (Figure 11D). Interestingly, *PPARγ* and *FABP-4* were subjected to a different regulation to what was observed when cells were differentiated directly for 14-days. Both were significantly downregulated in both concentrations of GMG-43AC (Figure 11E,F). These results suggest that GMG-43AC inhibited lipid accumulation in hADSCs in a dose-dependent manner and showed a potential prevention and treatment capability in these cells. Moreover, cell viability was studied by means of MTT assay and the results show that in comparison to control cells, demonstrating the lack of toxicity induced by the drug at 0.5 mM (Figure 12). However, we noticed toxicity in the 2 mM dosage of GMG-43AC.

## 3. Discussion

One strategy to reduce adiposity would be the reduction of adipocytes number and fat content by adipogenesis-inducing agents. In this study, post-confluent 3T3-L1 preadipocytes and hADSCs, were treated by adipocytes-inducing agents (IBMX, DEX and insulin [30]). We administrated the GMG-43AC drug at different dosages (0.3 mM, 0.5 mM, 1 mM, and 2 mM for the 3T3-L1 preadipocytes; 0.5 mM and 2 mM for hADSCs). The effects on the differentiation process were evaluated.

The Oil Red-O staining, used to investigate the efficiency of the differentiation, demonstrated a dose-dependent inhibition of the adipogenic process by the GMG-43AC drug. Furthermore, GMG-43AC treated cells resembled a fibroblast-like preadipocytes morphology in both 3T3-L1 and hADSCs, as opposed to the regular adipocytes shape, which is round and filled with lipid droplets. Moreover, cell viability assays showed that hADSCs were more sensitive to the GMG-43AC 2 mM dosage.

The differentiation of preadipocytes and hADSCs in adipocytes is regulated by a complex network of transcriptional factors. At the center of this network are *PPARγ*, and the members of the *C/EBP* family, which are important for adipogenesis. In the presence of hormonal stimulants, *C/EBPβ* and *C/EBPδ* levels increased rapidly and then synergistically stimulated the expression of *PPARγ* and *C/EBPα,* which cross-regulate each other through a positive feedback [6,9,10,11,31]. The exposure to GMG-43AC in 3T3-L1 during the initial 48 h of adipocyte differentiation significantly affects *C/EBPβ* and *C/EBPδ* levels in the 3T3-L1. However, we saw that lowest concentrations of GMG-43AC (0.3 or 0.5 mM) reduced *C/EBPβ* more than the higher concentrations. It is possible to see a more pronounced reduction in 0.3 mM and an increase with 1 mM of GMG-43AC. This suggests that GMG-43AC could act at another point in the adipogenic network, and we thus investigated its effect on *PPARγ* and *C/EBPα* We observed that at day 10 of the treatment, GMG-43AC inhibited both the expression of *PPARγ* and *C/EBPα* mRNA and proteins. Thus, we suggest that GMG-43AC inhibited adipogenesis by suppressing the expression of the transcriptional factors required for the differentiation process. This is in line with previous literature reporting the role of GMG-43AC as a selective *PPARγ* modulator [22]. The downstream target genes of *PPARγ* and *C/EBPα*, such as *FABP-4* and *leptin*, are adipocyte-specific genes involved in maintaining adipocyte phenotype. GMG-43AC also reduced the expression of *FABP-4* and *leptin*, which further supports its effect on the inhibition of adipogenesis through *PPARγ* modulation.

Lipolysis plays a central role in the regulation of energy balance. The control of lipolysis is complex and involves the hormone-sensitive lipase (*Hsl*), which is the rate-limiting enzyme responsible for mediating the hydrolysis of triglycerides [26]. Our results showed that exposure to GMG-43AC significantly reduced mRNA expression of *Hsl,* with a reduction of glycerol release. Moreover, we showed that after the removal of the drug from 3T3-L1, cells differentiated in adipocytes in the presence of GMG-43AC, and cells did not resume to synthesize and accumulate triglycerides, indicating that the inhibition of triglycerides synthesis was long term. Furthermore, the GMG-43AC drug was efficient in inhibiting adipogenesis even when cells were supplemented with troglitazone, a potent inducer of adipocytes differentiation.

We also demonstrate that the GMG-43AC inhibitory effect is effective also after the cells are fully differentiated, suggesting a promising strategy in the treatment of obesity. Furthermore, the drug’s effect is maintained when GMG-43AC is removed from the culture medium. For all the experiments, the effects were dosage sensitive, with the 1 mM and 2 mM dosages being the most effective in the inhibition of the differentiation in both 3T3-L1. This suggests a dosage-dependent mechanism, which was further investigated by leaving the 3T3-L1 cells in culture with GMG-43AC for 5 weeks. In this case, the 0.3 mM and the 0.5 mM also resulted in an efficient inhibition of adipogenesis, suggesting that a long-term exposure does not revert the phenotype and even makes lower dosages more effective.

Moreover, in hADSCs, in the 14-days differentiation process, both concentrations showed an inhibitory effect of *PPARγ* and *FABP-4*, contrary to the 10-days differentiation, when *FABP-4* was not significantly reduced in the 0.5 mM but was at 2 mM. *PPARγ* was significantly induced in the low concentration of 0.5 mM but significantly inhibited in the 2 mM. This discrepancy could be explained speculating an ago-antagonist mechanism for GMG-43AC or by specific modifications in the tree-dimensional conformation of the receptor induced by ligands that could lead to different possible transcriptional activities of *PPARγ* Interestingly, a number of selective *PPARγ* modulators that have distinct gene expression on profiles are being investigated for different indications [32,33,34]. Moreover, in different cellular contexts, such as sebocytes, *PPARγ* has been shown to inhibit lipid accumulation, thus suggesting a context-dependent mechanism [23,35]. We consider that 1 mM is a safe and effective dose in both types of cells, since in the 3T3 L1 cells the tunnel assay showed that the number of apoptotic nuclei was very low. hADSCs are more sensitive to the treatment as 0.5 mM of the drug efficiently inhibited the differentiation without toxicity. In vivo the dosages need to be adjusted. This may suggest that GMG-43AC could be effective in a dose sensitive manner.

## 4. Materials and Methods

**Figure 13:**
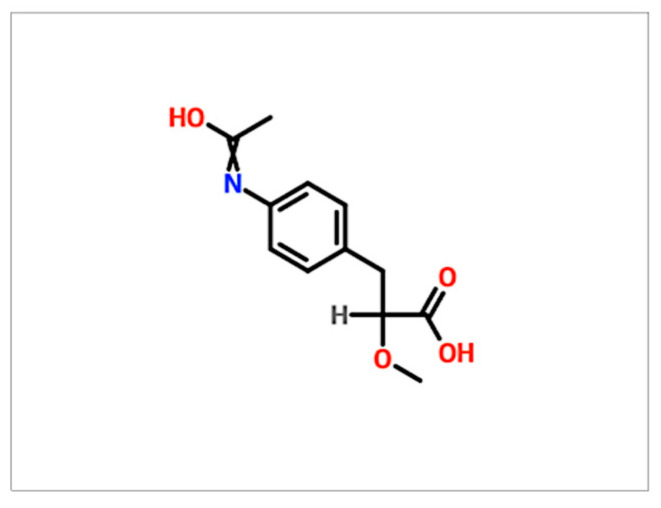
Molecular structure of the GMG-43AC molecule, as reported in the Molbase database.

Insulin (Sigma-Aldrich, St. Louis, MO, USA) was dissolved in HCl 0.005 N at the concentration of 10 mg/mL. Dexamethasone (DEX, Sigma-Aldrich) was dissolved in DMSO at the concentration of 1 mM. 3-isobutil-1-metylxantine (IBMX, Sigma-Aldrich) was dissolved in DMSO at the concentration of 0.5 M. Troglitazone (Sigma-Aldrich, St. Louis, MO, USA) was dissolved in DMSO at the concentration of 10 μM. GMG-43AC, was kindly given by Giuliani Sp.A. (Milan, Italy) and dissolved in DMSO at the concentration of 200 mM. The structure of the molecule is shown below (Figure 13) as reported in the Molbase database. The final concentration of DMSO added to the growth medium was less than 0.001%.

### 4.1. Cell Cultures and Induction of Differentiation in 3T3-L1 Cells

Mouse 3T3-L1 preadipocytes cells were obtained from the American Type Culture Collection (ATCC-CL-173). Briefly, preadipocytes were cultured in Dulbecco’s modified Eagle’s medium (DMEM) (Euro Clone S.p.A, Milan, Italy) containing 1 g/L d-glucose, 10% heat-inactivated fetal bovine serum (FBS) supplemented with antibiotics [25]. Cells were maintained at 37 °C in a humidified atmosphere containing 5% CO_2_. For preadipocytes’ differentiation, two days after confluence (defined as day 0), cells were exposed to the adipocyte differentiation medium (DMEM) containing 4.5 g/L d-glucose, 10% FBS, antibiotics and supplemented with dexamethasone (DEX; 1 µM), 3-isobutil-1-metylxantine (IBMX; 0.5 mM) and insulin (10 µg/mL) for 48 h. On day 2, the differentiation medium was substituted with DMEM containing insulin (10 µg/mL) for another 48 h. The last period of differentiation was conducted keeping the cells in differentiation maintenance medium composed of DMEM 4.5 g/L d-glucose with 10% FBS. The medium was changed every two days and the differentiation process lasted 10 days.

### 4.2. Cell Cultures and Induction of Differentiation in hADSCs Cells

Primary cell cultures from human adipose tissue samples were obtained from voluntary patients undergoing elective liposuction procedures under local anesthesia. The cells were isolated and expanded in a Biological Safety Cabinets (HERA Safe, HERAEUS, Newtown, CT, USA) and cells were cultured maintenance medium composed of Dulbecco’s modified Eagle’s medium (DMEM) (Euro Clone) containing 1 g/L d-glucose 10% heat-inactivated fetal bovine serum (FBS) supplemented with antibiotics at 37 °C in a humidified, 5% CO_2_ incubator (HERAcell 150-Thermo electron, Waltham, MA, USA) [24]. For adipogenic differentiation, two days after confluence (defined as day 0), cells were exposed to the adipocyte differentiation medium (DMEM) containing 4.5 g/L d-glucose, 10% FBS, antibiotics, and supplemented with dexamethasone (DEX; 1 µM), 3-isobutil-1-metylxantine (IBMX; 0.5 mM) and insulin (10 µg/mL). The medium was changed every two days and the differentiation process lasted 10 days or 14 days as specified in the Results section and figure legends.

### 4.3. Oil Red-O Staining and Quantification of Lipid Accumulation in Adipocytes

Lipid droplets in mature adipocytes or preadipocytes (control) were stained with Oil Red-O to determine the efficiency of differentiation. At the end of the observational period, cells were rinsed twice with phosphate buffered saline (PBS), fixed with 4% fresh formalin in PBS for 1 h, and rinsed twice with PBS. Afterwards, the cells were stained for 15 min with Oil Red-O (Sigma-Aldrich, 0.5 g in 100 mL isopropanol). The cells were then washed with distilled water to remove excess dye and photographed under microscopy. Nuclei were stained with hematoxylin (Sigma-Aldrich, St. Louis, Ma, USA) for 5 min at room temperature. Slides were captured using an optical microscope Leica and digital camera (Leica, Wetzlar, Germany). The quantification of positive cells was performed on a minimum of 9 independent fields (3 fields/3 coverslips/treatment) of photomicrographs captured with 20X objective. Total counts of positive cells were performed, and the number of positive cells was expressed as the percentage to the total cells (hematoxylin positive cells). To quantify the intracellular lipid accumulation of Oil Red-O, the stained lipid droplets were eluted with 100% isopropanol for 10 min. The optical density was measured at 500 nm by spectrophotometer.

### 4.4. Immunofluorescence in 3T3-L1 Cells

For the immunofluorescence experiments, cells were fixed with 2% and 4% paraformaldehyde in PBS and then permeabilized for 30 min with 0.4% Bovine Serum Albumin (BSA) and 0.03% Triton X-100 in PBS. The cells were incubated with antibodies directed against *anti-CCAAT/enhancer binding protein β (C/EBPβ)* (dilution 1:200; Santa Cruz Biotechnology, Dallas, Texas, USA); *C/EBPδ* (dilution 1:200; Santa Cruz); *PPARγ* (dilution 1:200), *C/EBPα* (dilution 1:50), *FABP-4* (dilution 1:200) and *leptin* (dilution 5 µg/mL, ABCAM, Cambridge, MA, USA), overnight at 4 °C. After washing with PBS, 0.04% BSA, 0.003% Triton-X100, cells were left for 45 min at room temperature with the secondary antibody Alexa Fluor 488 or 543 anti-rabbit or goat (Invitrogen, Carlsbad, CA, USA). The Nuclei were visualized after incubation with 0.2 mg/mL 4′,6′-diamidino-2-phenyl-indole (*DAPI*, Thermo-Fisher Scientific, Netherlands). In control experiments, primary antibodies were either omitted or replaced with equivalent concentrations of unrelated Ig of the same subclass. Slides were captured using a 40X objective lens and 1X zoom (Leica, Wetzlar, Germany) with confocal microscope Leica SP2 microscope with He/Kr and Ar lasers (Leica). The quantification of positive cells to investigated markers (see above) was performed by confocal microscopy. Cell counts were performed on a minimum of 9 independent fields (3 fields/3 coverslips/treatment) of photomicrographs captured with 40X objective. Total counts of each immunoreactive cell marker were performed and the number of positive cells per culture was expressed as the percentage to the total cells. DAPI supplied the total number of cells.

### 4.5. Western Blot Analysis

Cells were washed twice with cold PBS and lysed in RIPA buffer containing 40 mM TRIS-HCl (pH = 7.5), 150 mM NaCl, 1% Triton X-100, 0.1% SDS, 0.5% sodium deoxycholate, 0.1 mM EDTA and protease inhibitor cocktail. Protein concentration was measured at 595 nm by the Bradford method (Quick Start™ Bradford Protein Assay, BIO-RAD, Milan, Italy). Sixty micrograms of protein were separated by SDS-PAGE gel electrophoresis and transferred onto a nitrocellulose membrane. The membrane was blocked with 5% skim milk for 1 h and washed tree times with the TBS buffer with 0.05% Tween (T-TBS). The membrane was then incubated with primary antibodies diluted in T-TBS (1:1000). After incubation with the corresponding peroxidase-conjugated secondary antibodies IgG-HRP (Chemicon International, Tamecula, CA, USA) proteins were visualized using enhanced chemiluminescence detection system (ECL^TM^, Pierce, Fisher Scientific, 1120 AA Landsmeer, The Netherlands). After incubation with ECL, blots were placed in a sheet protector and exposed to Kodak X-Omat Blue Film (Blue X-ray Film) (Eastman-Kodak, Rochester, NY, USA for different times. After acquisition by a GelDoc^TM^ image capture system (Eastman-Kodak, Rochester, NY, USA), the autoradiograms were quantified using Quantity One^TM^ software. Primary antibodies used were *anti-peroxisome proliferator-activated receptor-γ* (*PPARγ*) (Cell Signaling Technology, Beverly, MA, USA); *anti-CCAAT/enhancer binding protein α (C/EBPα)* (Cell Signaling Technology Beverly, MA, USA); *anti-β-actin* (SIGMA, St. Louis, MO, USA).

### 4.6. Real-Time PCR Analysis

Total RNA was isolated by using TRI^®^ Reagent (Sigma-Aldrich, St. Louis, MO, USA) in accordance with the manufacturer’s instructions. The synthesis of single-strand cDNA was carried out on 1 μg of RNA, using iScript™ Reverse Transcription Supermix (BIO-RAD, Milan, Italy) following manufacturer’s instructions. Real-time PCR was performed with the StepOnePlus^TM^ Real-Time PCR System (Thermo Fisher, Carlsbad, CA, USA) using iQ SYBR Green Supermix (BIO-RAD, Milan, Italy). A non-template control (NTC) was added in order to exclude the presence of genomic DNA, and all experiments were performed in triplicates. Samples were analyzed with the 2^ΔΔCt^ method. The mRNA levels of target genes were normalized with 18S ribosomal RNA for 3T3-L1 cells, and *GAPDH* for hADSCs. The primers were designed using Oligo Perfect^®^ Designer Software (Invitrogen). The nucleotide sequences of the primers utilized are reported in Table 1 and Table 2.

### 4.7. Lipolysis Measurement

Lipolysis was evaluated by measuring the amount of glycerol released into the growth medium. Lipolysis was assayed after 2, 4, and 10 days of GMG43AC treatment by means of a commercial Assay KIT for Glycerol Detection (Zen-Bio Inc., Research Triangle Park, NC, USA) according to the manufacturer’s instructions. The increase in absorbance at 540 nm was directly proportional to glycerol concentration of the sample and was measured in spectrophotometer Bio UV/VIS^®^ Parkin Helmer (Buckinghampshire, UK).

### 4.8. Cell Viability and Terminal deoxynucleotidyl transferase dUTP Nick end Labeling Assay

Cell viability was determined by the exclusion of propidium iodide 0.5 mg/mL in culture after a 30 minutes’ incubation [36]. Terminal deoxynucleotidyl transferase dUTP nick end labeling (TUNEL) was used for detecting DNA fragmentation. The DeadEnd™ Fluorometric TUNEL System (Promega, Madison, WI, USA) was used and the assay was performed after 10 days of incubation of cells with or without GMG-43AC.

### 4.9. MTT Assay

Cell viability was measured by a quantitative colorimetric MTT assay, sensitive of the metabolic statuses of cells, particularly the mitochondrial status, thus reflecting early redox changes. Briefly, hADSCs were seeded in a 96-well plate at a density of 2 × 10^3^ cells/well. After the cellular treatments were over, 10 μL of MTT assay kit reagent (Sigma-Aldrich, St. Louis, MO, USA) was added to each well, and the cells were incubated for an additional three hours. MTT was eluted with a solution containing HCl 4 mM, 0.1% NP40, Isopropanol for 30 min. The absorbance of each reaction product was measured with EnSight™ multimode plate reader (PerkinElmer, Waltham, MA, USA) at a wavelength of 560 nm. The results are expressed as a percentage of the MTT absorbance of the control cells, which was set to 100%.

### 4.10. Statistical Analysis

All data were expressed as mean ± SEM. Multiple comparisons were analyzed by one-way analysis of variance (ANOVA) followed by Turkey post hoc test. Statistical significance was accepted at a level *p* < 0.05.

## 5. Conclusions

Our data demonstrates that the treatment with GMG-43AC inhibits both 3T3-L1 preadipocytes and hADSCs differentiation most likely by the suppression of *PPARγ* and *C/EBPα.* This leads to the inhibition of downstream molecules *FABP-4* and *leptin*, which are also fundamental markers in the regulation of the adipogenesis process. Consequently, the inhibition of adipogenesis may regulate the lipolysis process. Furthermore, in vitro analysis of hADSCs differentiation helped the translation of these findings to humans which showed an initial and a promising approach in the prevention of adipogenesis.

Further studies are needed to evaluate the beneficial effects of GMG-43AC on adipogenesis and preclinical murine models of the disease will be needed to evaluate the in vivo effects of the molecule.

## Figures and Tables

**Table 1:** Primer sequences for Mouse 3T3L1 cells.

Gene Name	Forward Primer	Reverse Primer
*C/EBP*β	CGCCTACCTGGGCTACCA	GACAGCTGCTCCACCTTCTTC
*C/EBP*δ	ATACCTCAGACCCCGACAGC	ATGCTTTCCCGTGTTCCTTC
*C/EBP*α	GAAGGTGCTGGAGTTGACCA	AGGAAGCAGGAATCCTCCAA
*PPAR*γ	GTGGGGATGTCTCACAATGC	TGATCTCTTGCACGGCTTCT
*FABP*-4	ACGGCCCTGCAGAACTATCT	AAGGTTCACAAACGCGACAG
*leptin*	TGTGCACCTGAGGGTAGAGG	CCCTGGACAACCTTGGAGAT
*Hsl*	GCTTCTCCCTCTCGTCTGCT	CAGACACACTCCTGCGCATA
*18S rRNA*	ACCGCGGTTCTATTTTGTTG	GACAAATCGCTCCACCAACT

**Table 2:** Primer sequences for human Adipose Derived Mesenchymal Cells.

Gene Name	Forward Primer	Reverse Primer
*PPAR* *γ*	CAAGAGTACCAAAGTGCAATCAAAGTGGAG	GTTCTCCGGAAGAAACCCTTGCATCCTTCA
*FABP-4*	CTGGGCCAGGAATTTGACGA	ACCAGGACACCCCATCTAA
*GAPDH*	CTTTTGCGTCGCCAG	TTGATGGCAACAATATCCAC

## Abbreviations

hADSCs	human Adipose-Derived Stem Cells
PA	Propanoic Acid
BMI	Body Mass Index
*C/EBP*	*CCAAT*/enhancer-binding protein *α,* β, δ
*PPAR γ*	Peroxisome proliferator-activated receptor γ
*FABP-4*	Fatty acid binding protein
HSL	Hormone-sensitive lipase gene
TUNEL	Terminal deoxynucleotidyl transferase dUTP nick end labeling
TZD	Troglitazone
DEXA	Dexamethasone
DMSO	Dimethyl Sulfoxide; IBMX isobutil-1-metylxantine
DAPI	4′,6′-diamidino-2-phenyl-indole
FBS	Fetal bovine serum
NHK	normal human keratinocytes
ECM	Extracellular Matrix
NP-40	Nonidet P-40
MSC	Mesenchymal stem cells
HCL	Hydrogen Chloride
DMEM	Dulbecco’s modified Eagle’s medium
PBS	Phosphate Buffer Solution
BSA	Bovine Serum Albumin
